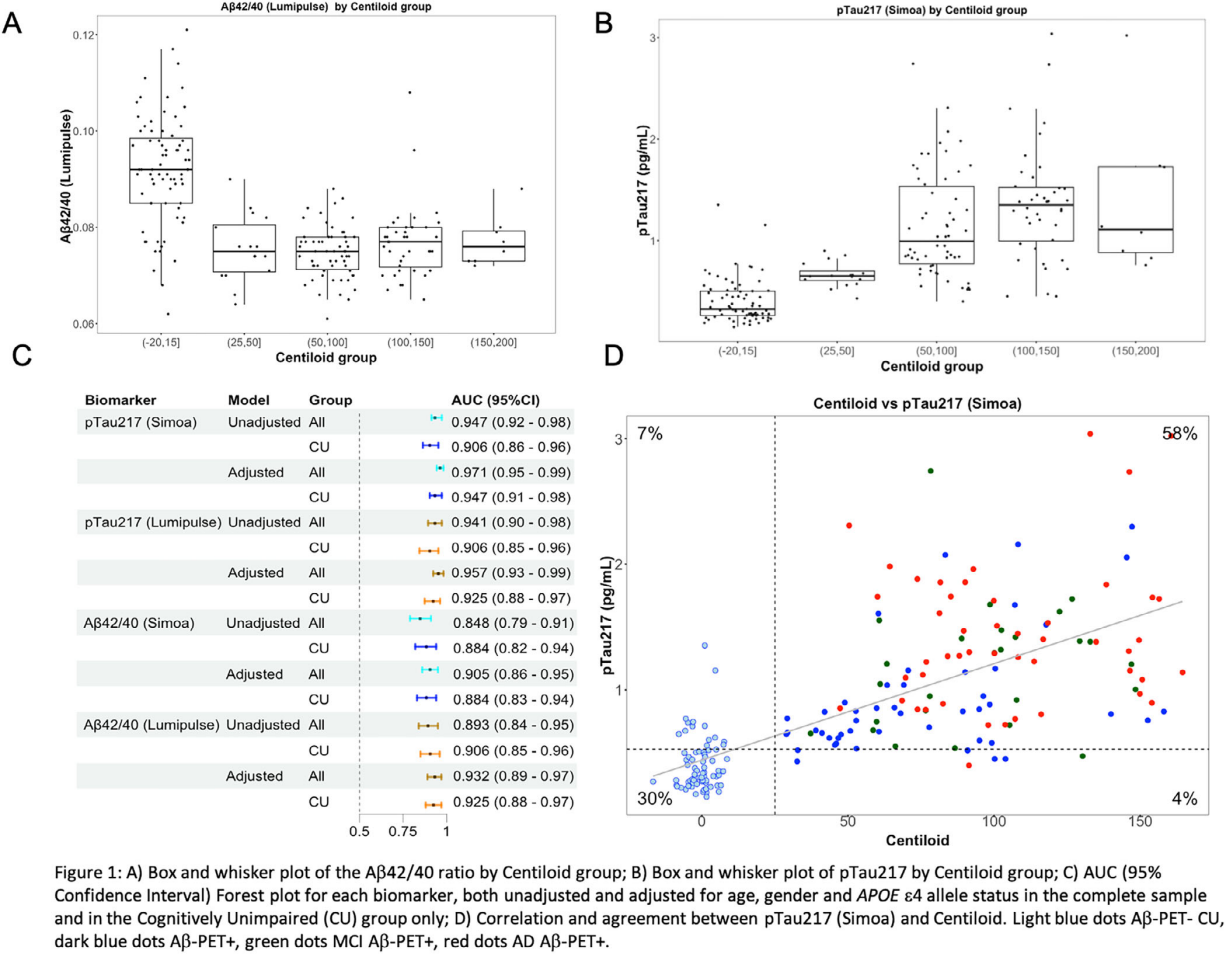# Comparing plasma Aβ42/40 and pTau217 to predict Amyloid‐β status across the Alzheimer’s disease continuum

**DOI:** 10.1002/alz.091746

**Published:** 2025-01-09

**Authors:** James D. Doecke, Ahmed Chenna, Mintzu Lo, Youssouf Badal, Brandon Yee, Robert L. Martone, Christos Petropoulos, Christopher J Fowler, Simon M. Laws, Stephanie Rainey Smith, Ralph N Martins, Christopher C. Rowe, Colin L Masters, John W Winslow

**Affiliations:** ^1^ School of Medical and Health Sciences, Edith Cowan University, Perth, Western Australia Australia; ^2^ The Australian e‐Health Research Centre, CSIRO, Brisbane, QLD Australia; ^3^ Monogram Biosciences/Labcorp, South San Francisco, CA USA; ^4^ Labcorp‐Monogram Biosciences, South San Francisco, CA USA; ^5^ Labcorp Drug Development, Indianapolis, IN USA; ^6^ The Florey Institute of Neuroscience and Mental Health, The University of Melbourne, Parkville, VIC Australia; ^7^ Centre for Precision Health, Edith Cowan University, Joondalup, Western Australia Australia; ^8^ Centre for Healthy Ageing, Murdoch University, Murdoch, Western Australia Australia; ^9^ Centre of Excellence for Alzheimer’s Disease Research and Care, School of Medical and Health Sciences, Edith Cowan University, Joondalup, Western Australia Australia; ^10^ Australian Alzheimer's Research Foundation, Nedlands, Western Australia Australia; ^11^ School of Psychological Science, University of Western Australia, Crawley, Western Australia Australia; ^12^ Department of Biomedical Sciences, Macquarie University, Macquarie Park, NSW Australia; ^13^ Department of Nuclear Medicine and Centre for PET, Austin Health, Heidelberg, Vic, 3084, Australia, Heidelberg, VIC Australia

## Abstract

**Background:**

Recent advances in immunoassays have enabled sensitive detection of Aβ42/40 and pTau217 in plasma, components of Alzheimer’s disease (AD) neuropathological markers. Further characterization of increased diagnostic accuracy with PET Amyloid‐β (Aβ) across the AD continuum is needed for clinical application.

**Method:**

Participants from the Australian Imaging, Biomarkers and Lifestyle (AIBL) study of ageing (N=197) representing a cross‐sectional population of four clinical and PET‐Aβ subgroups: cognitive unimpaired (CU) Aβ‐ (n=75), CU Aβ+ (n=48), mild cognitive impairment (MCI) Aβ+ (n=26), and AD Aβ+ (n=48). EDTA plasma was analyzed with Aβ42/40 (Quanterix Simoa & Fujirebio Lumipulse) and pTau217 (ALZpath Simoa & Lumipulse). Data were investigated using Cohen’s D for effect size, Receiver Operating Characteristic (ROC) analyses to define AUC values for PET‐Aβ positivity and Spearman’s Rho for correlation between biomarkers and Centiloid.

**Result:**

Lower Lumipulse and Simoa plasma Aβ42/40 ratios were observed in Aβ+ vs Aβ‐ groups (p<0.0001; with effect size Cohen’s D indices = 1.39 and 1.07, respectively). Aβ42/40 ratios initially decreased with increasing amyloid PET Centiloid levels through the positivity threshold (25CL), levelling off with increasing Centiloid values across the disease continuum (Figure 1A). Highest effects sizes were seen for pTau217 when comparing Aβ+ vs Aβ‐ groups (p<0.0001; Cohen’s D: 1.54 [Simoa] and 1.49 [Lumipulse]), with stepwise increases per Centiloid group (Figure 1B). Biomarker AUC values to predict PET‐Aβ were highest for pTau217 in the complete sample compared with the CU sample (Simoa AUC: 0.947/0.906, Lumipulse AUC: 0.941/0.906). For the Aβ42/40 ratio, AUC values were higher in the CU group as compared with the complete sample (Lumipulse AUC: 0.896/0.889, Simoa AUC: 0.885/0.849 (Figure 1C). Adding in age, gender, and APOE ε4 allele status improved prediction of PET‐Aβ, with AUC values reaching 0.97 for pTau217 (Figure 1C). Correlations between biomarker and Centiloid were higher for pTau217 (Simoa: r=0.744 (Figure 1D), Lumipulse r=0.732) as compared with the Aβ42/40 ratio (Lumipulse r=‐0.509, Simoa r=‐0.462).

**Conclusion:**

Sensitive assays for the Aβ42/40 ratio may be more appropriate to detect Aβ burden in CU participants while p‐Tau217 appears to be more accurate and has the highest effect size and AUC values to predict Aβ‐PET across the AD continuum.